# Supplementary material for: Polyketide synthase gene clusters in the ericoid mycorrhizal fungus Oidiodendron maius: genome-wide prediction, expression profiling, and functional characterization of knockout mutants
Source: Front Plant Sci. 2026 Sep 16;17:1924430. doi: 10.3389/fpls.2026.1924430 (PMC13623903; doi:10.3389/fpls.2026.1924430)
Supplement: Supplementary file 1 [file SupplementaryFile1.zip › Supplementary Materials/Supplementary Folder 1.zip!/Oidma1_AssemblyScaffolds_Repeatmasked/knownclusterblast/cluster36/jgi.p_Oidma1_32887_mibig_hits.html]

| MIBiG Protein | Description | MIBiG Cluster | MiBiG Product | % ID | % Coverage | BLAST Score | E-value |
| --- | --- | --- | --- | --- | --- | --- | --- |
| BAE56588.1 |  | BGC0001123 | Aspirochlorine\_biosynthetic\_gene\_cluster | 52 | 104.798464491 | 552 | 4e-157 |
| EAQ86388.1 | hypothetical\_protein | BGC0001405 | Chaetoviridin\_/\_chaetomugilin\_biosynthetic\_gene\_cluster | 53 | 102.111324376 | 519 | 4.9e-147 |
| ADI24948.1 | GsfI | BGC0000070 | Griseofulvin\_biosynthetic\_gene\_cluster | 48 | 100.959692898 | 479 | 5.6e-135 |
| ACD39771.1 | non-heme\_halogenase | BGC0000134 | Radicicol\_biosynthetic\_gene\_cluster | 45 | 103.071017274 | 453 | 2.5e-127 |
| AEM76785.1 | putative\_halogenase | BGC0001401 | Melleolides\_biosynthetic\_gene\_cluster | 46 | 102.879078695 | 451 | 9.6e-127 |
| AGO59046.1 | PtaM | BGC0000121 | Pestheic\_acid\_biosynthetic\_gene\_cluster | 43 | 102.879078695 | 451 | 9.6e-127 |
| ALT31850.1 | ArmH3 | BGC0001401\_c3 | Melleolides\_biosynthetic\_gene\_cluster | 47 | 100.19193858 | 445 | 8.9e-125 |
| AEM76786.1 | putative\_halogenase | BGC0001401\_c2 | Melleolides\_biosynthetic\_gene\_cluster | 45 | 101.727447217 | 445 | 8.9e-125 |
| ALT31851.1 | ArmH4 | BGC0001401\_c4 | Melleolides\_biosynthetic\_gene\_cluster | 44 | 104.222648752 | 421 | 1.8e-117 |
| ALT31852.1 | ArmH5 | BGC0001401\_c5 | Melleolides\_biosynthetic\_gene\_cluster | 42 | 101.919385797 | 410 | 4.2e-114 |
| ADE22292.1 | putative\_non-heme\_halogenase | BGC0000279 | Xantholipin\_biosynthetic\_gene\_cluster | 37 | 75.0479846449 | 269 | 8.8e-72 |
| ABM21576.1 | CrpH | BGC0000975 | Cryptophycin\_biosynthetic\_gene\_cluster | 32 | 102.687140115 | 265 | 9.7e-71 |
| AMK51270.1 | Hex13 | BGC0001376 | Hexaricin\_biosynthetic\_gene\_cluster | 35 | 85.0287907869 | 255 | 1.7e-67 |
| CAJ46693.1 | tryptophan\_halogenase | BGC0000969 | Chondramide\_biosynthetic\_gene\_cluster | 37 | 77.5431861804 | 253 | 6.5e-67 |
| CAM34371.1 | putative\_non-heme\_halogenase | BGC0000242 | Lysolipin\_biosynthetic\_gene\_cluster | 33 | 89.8272552783 | 248 | 1.6e-65 |
| ACU71639.1 | tryptophan\_halogenase | BGC0001154 | Cacibiocin\_biosynthetic\_gene\_cluster | 31 | 76.1996161228 | 215 | 1.2e-55 |
| WP\_052390547.1 | halogenase | BGC0001308 | Simocyclinone\_D9\_/\_simocyclinone\_D10\_/\_simocyclinone\_D11 | 31 | 74.8560460653 | 209 | 1.1e-53 |
| AEU17894.1 | putative\_halogenase | BGC0001072 | Simocyclinone\_biosynthetic\_gene\_cluster | 31 | 74.8560460653 | 208 | 2.4e-53 |
| CAA76550.1 | halogenase | BGC0000311 | Balhimycin\_biosynthetic\_gene\_cluster | 30 | 95.009596929 | 191 | 3e-48 |
| AAM80532.1 | StaI | BGC0000290 | A47934\_biosynthetic\_gene\_cluster | 31 | 87.3320537428 | 190 | 6.8e-48 |
| AEI58871.1 | halogenase | BGC0000455 | Vancomycin\_biosynthetic\_gene\_cluster | 30 | 95.009596929 | 188 | 1.5e-47 |
| AGS77325.1 | Halogenase | BGC0001178 | UK-68,597\_biosynthetic\_gene\_cluster | 30 | 95.3934740883 | 188 | 1.5e-47 |
| AAK81830.1 | non-heme\_halogenase | BGC0000326 | Complestatin\_biosynthetic\_gene\_cluster | 31 | 77.7351247601 | 188 | 2.6e-47 |
| ADU85999.1 | putative\_tryptophan\_halogenase | BGC0000165 | Tiacumicin\_B\_biosynthetic\_gene\_cluster | 29 | 83.3013435701 | 186 | 5.7e-47 |
| AGC09515.1 | FAD\_dependent\_halogenase | BGC0001183 | Lobophorin\_biosynthetic\_gene\_cluster | 32 | 77.1593090211 | 185 | 1.3e-46 |
| CAG15020.1 | halogenase | BGC0000441 | Teicoplanin\_biosynthetic\_gene\_cluster | 30 | 95.009596929 | 184 | 2.8e-46 |
| CAE53362.1 | halogenase | BGC0000440 | Teicoplanin\_biosynthetic\_gene\_cluster | 30 | 95.009596929 | 184 | 2.8e-46 |
| CAA11780.1 | PCZA361.26 | BGC0000322\_c2 | Chloroeremomycin\_biosynthetic\_gene\_cluster | 29 | 95.009596929 | 183 | 6.3e-46 |
| AAN65237.1 | putative\_halogenase | BGC0000832 | Clorobiocin\_biosynthetic\_gene\_cluster | 33 | 68.905950096 | 181 | 3.1e-45 |
| AFV52210.1 | aryl-ACP\_halogenase | BGC0000081 | Kedarcidin\_biosynthetic\_gene\_cluster | 30 | 86.3723608445 | 180 | 5.4e-45 |
| CAD91205.1 | halogenase | BGC0000289 | A40926\_biosynthetic\_gene\_cluster | 31 | 77.5431861804 | 178 | 2e-44 |
| AAL06656.1 | alkylhalidase | BGC0000965 | C-1027\_biosynthetic\_gene\_cluster | 30 | 77.9270633397 | 172 | 1.5e-42 |
| ABY66006.1 | halogenase | BGC0001008 | Maduropeptin\_biosynthetic\_gene\_cluster | 30 | 77.7351247601 | 170 | 4.3e-42 |
| ctg1\_orf12 |  | BGC0001366 | BE-24566B\_/\_zunyimycin\_A\_biosynthetic\_gene\_cluster | 32 | 76.0076775432 | 170 | 7.3e-42 |
| ALU98528.1 | tryptophan\_halogenase | BGC0001397 | Lidamycin\_biosynthetic\_gene\_cluster | 31 | 76.3915547025 | 169 | 9.5e-42 |
| ALD82533.1 | halogenase | BGC0001212 | Nannocystin\_biosynthetic\_gene\_cluster | 31 | 77.3512476008 | 167 | 4.7e-41 |
| ABD65950.1 | halogenase | BGC0000341 | Enduracidin\_biosynthetic\_gene\_cluster | 31 | 77.1593090211 | 165 | 2.3e-40 |
| ABS50460.1 | NapH2 | BGC0001079 | Napyradiomycin\_biosynthetic\_gene\_cluster | 30 | 70.8253358925 | 162 | 1.5e-39 |
| ABS50488.1 | NapH2 | BGC0000652 | Napyradiomycin\_biosynthetic\_gene\_cluster | 30 | 70.8253358925 | 162 | 1.5e-39 |
| AGS77327.1 | Halogenase | BGC0001178 | UK-68,597\_biosynthetic\_gene\_cluster | 30 | 80.6142034549 | 161 | 3.4e-39 |
| CAQ43074.1 | halogenase | BGC0000970 | Chondrochloren\_biosynthetic\_gene\_cluster | 31 | 77.5431861804 | 161 | 3.4e-39 |
| ADM46362.1 | halogenase | BGC0000106 | Naphthomycin\_biosynthetic\_gene\_cluster | 30 | 74.472168906 | 158 | 1.7e-38 |
| AAM54090.1 | halogenase | BGC0000020 | Ansamitocin\_biosynthetic\_gene\_cluster | 31 | 74.472168906 | 150 | 4.6e-36 |
| AFP87533.1 | FADH2-dependent\_halogenase | BGC0001159 | Marinopyrrole\_biosynthetic\_gene\_cluster | 30 | 71.4011516315 | 145 | 1.5e-34 |
| AAM80530.1 | StaK | BGC0000290 | A47934\_biosynthetic\_gene\_cluster | 30 | 74.2802303263 | 143 | 5.6e-34 |
| AAM70353.1 | CalO3 | BGC0000033 | Calicheamicin\_biosynthetic\_gene\_cluster | 28 | 75.4318618042 | 135 | 2e-31 |
| ESP90845.1 | Dehydrogenases\_(flavoprotein) | BGC0000891 | Bromophenols\_/\_bromopyrroles\_biosynthetic\_gene\_cluster | 26 | 67.754318618 | 118 | 2.5e-26 |
| AHA34035.1 | Bmp2 | BGC0000890 | Bromophenols\_/\_bromopyrroles\_biosynthetic\_gene\_cluster | 26 | 67.754318618 | 114 | 4.7e-25 |
